# A New Nomogram for Predicting the Risk of Intracranial Hemorrhage in Acute Ischemic Stroke Patients After Intravenous Thrombolysis

**DOI:** 10.3389/fneur.2022.774654

**Published:** 2022-03-10

**Authors:** Ze-An Weng, Xiao-Xiong Huang, Die Deng, Zhen-Guo Yang, Shu-Yuan Li, Jian-Kun Zang, Yu-Feng Li, Yan-Fang Liu, You-Sheng Wu, Tian-Yuan Zhang, Xuan-Lin Su, Dan Lu, An-Ding Xu

**Affiliations:** ^1^Department of Neurology and Stroke Center, The First Affiliated Hospital of Jinan University, Jinan University, Guangzhou, China; ^2^Clinical Neuroscience Institute, The First Affiliated Hospital of Jinan University, Jinan University, Guangzhou, China; ^3^Department of Neurology and Stroke Center, The Central Hospital of Shaoyang, Shaoyang, China

**Keywords:** acute ischemic stroke, intravenous thrombolysis, intracranial hemorrhage, nomogram, predictive model

## Abstract

**Background:**

We aimed to develop and validate a new nomogram for predicting the risk of intracranial hemorrhage (ICH) in patients with acute ischemic stroke (AIS) after intravenous thrombolysis (IVT).

**Methods:**

A retrospective study enrolled 553 patients with AIS treated with IVT. The patients were randomly divided into two cohorts: the training set (70%, *n* = 387) and the testing set (30%, *n* = 166). The factors in the predictive nomogram were filtered using multivariable logistic regression analysis. The performance of the nomogram was assessed based on the area under the receiver operating characteristic curve (AUC-ROC), calibration plots, and decision curve analysis (DCA).

**Results:**

After multivariable logistic regression analysis, certain factors, such as smoking, National Institutes of Health of Stroke Scale (NIHSS) score, blood urea nitrogen-to-creatinine ratio (BUN/Cr), and neutrophil-to-lymphocyte ratio (NLR), were found to be independent predictors of ICH and were used to construct a nomogram. The AUC-ROC values of the nomogram were 0.887 (95% CI: 0.842–0.933) and 0.776 (95% CI: 0.681–0.872) in the training and testing sets, respectively. The AUC-ROC of the nomogram was higher than that of the Multicenter Stroke Survey (MSS), Glucose, Race, Age, Sex, Systolic blood Pressure, and Severity of stroke (GRASPS), and stroke prognostication using age and NIH Stroke Scale-100 positive index (SPAN-100) scores for predicting ICH in both the training and testing sets (*p* < 0.05). The calibration plot demonstrated good agreement in both the training and testing sets. DCA indicated that the nomogram was clinically useful.

**Conclusions:**

The new nomogram, which included smoking, NIHSS, BUN/Cr, and NLR as variables, had the potential for predicting the risk of ICH in patients with AIS after IVT.

## Introduction

Stroke is the second leading cause of death and a major leading contributor to disability worldwide (1). Intravenous thrombolysis (IVT) therapy with recombinant tissue plasminogen activator (rt-PA) has proven to be a beneficial treatment for acute ischemic stroke (AIS) patients when administered within 4.5 h after stroke onset (2, 3). However, IVT therapy carries an inherent risk of complications, such as intracranial hemorrhage (ICH), that are associated with poor clinical outcomes. Furthermore, symptomatic intracranial hemorrhage (symptomatic ICH) is the most serious complication of IVT (4, 5).

Since ICH may lead to poor prognosis, a reliable scoring tool that can be used to identify the risk of post-thrombolysis ICH is essential. Several scoring systems have been applied to predict the risk of ICH after thrombolysis (6–10). However, most of these systems convert continuous variables to categorical variables, and this change might result in loss of information (11, 12). In addition, several prognostic nomograms for ICH events in patients with AIS who underwent thrombolysis have been reported, most of which were constructed based on Western populations (13–15). It was reported that the rate of ICH in Asian populations was 10.6%, while the rate of ICH in non-Asian populations was reported to be 4.74% (16). The reported ICH occurrence rate is 2.12-fold higher in Asian populations than in non-Asian populations. Two nomogram models based on Asian populations have been designed (12, 17). However, those nomogram models only include demographic and clinical factors, such as hypertension, blood glucose, blood platelet (PLT), and National Institutes of Health of Stroke Scale (NIHSS) scores, and ignore other factors that can be assessed based on laboratory examinations and that may be important risk factors for predicting ICH. We wondered whether some parameters that can be measured through laboratory examination could be risk factors for ICH.

In this study, we aimed to develop a new nomogram to predict the risk of ICH after IVT therapy and to compare the performance of our model with that of other scoring systems for predicting ICH. The nomogram we developed will help clinicians identify patients with AIS with a higher risk of ICH after IVT therapy.

## Methods

### Study Population and Design

The present study was a retrospective study performed in the stroke center of the Central Hospital of Shaoyang between November 2013 and January 2021. The patients met the following inclusion criteria: 1) more than 18 years of age; 2) diagnosed with AIS confirmed by MRI within 24 h after admission; 3) onset-to-treatment time for thrombolysis of <4.5 h; 4) they or their legal representatives provided written informed consent to participation in the study; and 5) treated with IVT. The exclusion criteria were as follows: 1) treated with endovascular procedures after IVT; and) not treated with IVT or refused IVT treatment; and 3) medical contraindications for IVT.

This study was approved by the Ethics Committee of the Central Hospital of Shaoyang. The Ethics Committee approval letter is available in Online Resource 1.

### Baseline Data Collection

We collected demographic, clinical, and laboratory information at admission and during hospitalization. The baseline data included age, sex, current smoking status, current drinking status, NIHSS score on admission, systolic blood pressure (SBP), diastolic blood pressure (DBP), blood glucose level on admission, history of hypertension and diabetes mellitus, atrial fibrillation (AF), international normalized ratio (INR), activated partial thromboplastin time (APTT), PLT, fibrinogen, albumin (ALB), white blood cells (WBC), neutrophil-to-lymphocyte ratio (NLR), high-density lipoprotein (HDL), low-density lipoprotein (LDL), triglyceride (TG), total cholesterol (TC), blood urea nitrogen-to-creatinine ratio (BUN/Cr), and uric acid (UA).

### Thrombolysis Method

All patients received rt-PA treatment within 4.5 h after the onset of stroke. Intravenous rt-PA at 0.9 mg/kg (90 mg maximum) was used; 10% of the total dose was administered as an intravenous bolus, followed by infusion of the remaining dose over 60 min (18).

### Ascertainment of ICH

All patients underwent a CT scan on admission, and each patient underwent another CT scan 24 h after intravenous rt-PA administration. In cases in which the patient's neurological function deteriorated rapidly, a CT scan was immediately performed to assess the presence of ICH. According to the European Cooperative Acute Stroke Study-2 criteria (19), the discovery of blood at any site in the brain on a CT scan indicated the presence of ICH. Symptomatic ICH was defined as any type of ICH accompanied by an increase in the NIHSS score by 4 or more points from baseline or leading to death; asymptomatic ICH was defined as any type of ICH without neurological deterioration. The endpoint in our research was ICH, including symptomatic ICH and asymptomatic ICH.

### Statistical Analysis

A total of 553 patients were randomly divided into two groups, the training set (*n* = 387) and the testing set (*n* = 166), at a theoretical ratio of 7:3. Categorical variables and continuous variables are expressed as frequencies (percentages, %) and means (SDs) or medians (interquartile ranges, IQRs), respectively. The differences in baseline characteristics between the training set and the testing set were assessed using Student's *t*-test or the non-parametric Mann–Whitney U test for continuous variables and the χ^2^ or Fisher's exact test for categorical variables. The least absolute shrinkage and selection operator (LASSO) method was used to screen the optimal risk factors related to ICH in the total dataset. The variables with non-zero coefficients in the LASSO regression model were selected for further analysis. In the training cohort, univariable logistic regression analysis was used to predict the probability of ICH. The variables with *P*-value <0.05 were added to the multivariable logistic regression analysis to screen for independent clinical predictors related to ICH. A nomogram was generated based on these risk factors in multivariable analysis. The area under the receiver operating characteristic curve (AUC-ROC) analysis was performed to assess the predictive accuracy of the nomogram, and calibration curves were drawn to compare the predicted probabilities with the observed probabilities. Decision curve analysis (DCA) was used to evaluate the clinical value of the predictive model.

The overall predictive and discriminative performance of the nomogram was compared with that of the Multicenter Stroke Survey (MSS) score, the Glucose, Race, Age, Sex, Systolic blood Pressure, and Severity of stroke (GRASPS) score, and the stroke prognostication using age and NIH Stroke Scale-100 positive index (SPAN-100) score. The AUC-ROC was used to estimate the accuracy and discrimination of the nomogram and these scoring systems in the training and testing sets. DeLong's test was performed to compare the differences in AUC-ROC between the ICH nomogram and the MSS score, GRASPS score, and SPAN-100 score.

Statistical analyses were performed using R version 3.6.3 software (http://www.R-project.org/) and SPSS version 27.0 (IBM, New York, NY, USA). Two-tailed values of *p* < 0.05 were considered statistically significant.

## Results

### Baseline Characteristics

**Figure 1:**
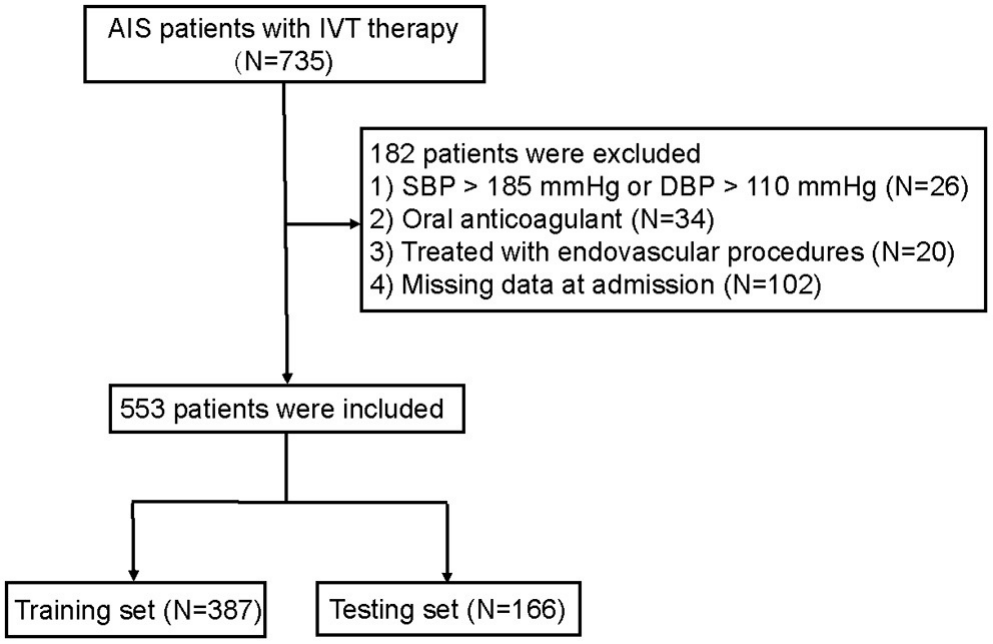
Flow diagram of the selection of eligible patients. AIS, acute ischemic stroke; IVT, intravenous thrombolysis. SBP, systolic blood pressure; DBP, diastolic blood pressure.

A total of 735 patients with AIS were initially enrolled in this study between November 2013 and January 2021. Of these, 182 patients who met the exclusion criteria were removed from the study, and 553 patients were finally eligible for analysis (Figure 1).

The training set consisted of 387 individuals, and the remaining 166 individuals were included in the testing set. The baseline characteristics of the patients in the training and testing sets are shown in Table 1. The median age of the patients in the training set was 69.51 ± 12.09 years, and 253 (65.37%) of them were men. The testing set consisted of 111 men (66.87%) with a median age of 68.73 ± 12.21 years. Of the total patients, the overall number with ICH was 51 (9.22%). The percentages of patients with ICH were 8.01 and 12.65% in the training set and the testing set, respectively. All variables were balanced between the two groups (*p* > 0.05).

**Table 1:** Baseline characteristics of AIS patients with IVT in the training and testing sets.

**Variables**	**Total sample (*n =* 553)**	**Training set (*n =* 387)**	**Testing set (*n =* 166)**	***P*-value**
ICH, *n* (%)	51(9.22)	31(8.01)	21(12.65)	0.120
**Demographic characteristics**				
Age, years	68.28 ± 12.12	69.51 ± 12.09	68.73 ± 12.21	0.491
Gender (male), *n* (%)	364(65.82)	253(65.37)	111(66.87)	0.809
**Clinical parameters**				
NHISS, score	8.30 ± 7.02	8.03 ± 6.76	8.94 ± 7.57	0.182
Current smoking, *n* (%)	84(15.19)	64(16.54)	20(12.05)	0.223
Current drinking, *n* (%)	44(7.96)	33(8.53)	11(6.63)	0.558
SBP, mmHg	151.52 ± 21.37	151.53 ± 21.42	151.47 ± 21.34	0.974
DBP, mmHg	86.13 ± 12.81	86.25 ± 12.51	85.87 ± 13.52	0.757
Blood glucose, mmol/L	7.57 ± 2.94	7.51 ± 2.96	7.70 ± 2.89	0.476
**Pre-existing comorbidities**				
Hypertension, *n* (%)	298(53.89)	205(52.97)	93(56.02)	0.571
Diabetes, *n* (%)	89(16.09)	59(15.25)	30(18.07)	0.482
History of stroke, *n* (%)	68(12.30)	49(12.66)	19(11.45)	0.797
AF, *n* (%)	62(11.21)	40(10.34)	22(13.25)	0.396
**Laboratory parameters**				
INR,	0.98 ± 0.11	0.98 ± 0.10	0.99 ± 0.12	0.684
APTT, s	27.45 ± 5.24	27.36 ± 5.24	27.65 ± 5.25	0.565
PLT, 10∧9/L	199.37 ± 57.52	200.98 ± 57.49	195.63 ± 57.58	0.317
Fibrinogen, g/L	2.74 ± 0.67	2.70 ± 0.6470	2.83 ± 0.73	0.043
ALB, g/L	40.70 ± 3.93	40.88 ± 3.99	40.29 ± 3.78	0.104
WBC, 10∧9/L	8.55 ± 3.09	8.46 ± 3.04	8.77 ± 3.19	0.285
NLR	5.15 ± 3.60	5.22 ± 3.66	5.01 ± 3.49	0.521
HDL, mmol/L	1.20 ± 0.35	1.21 ± 0.35	1.17 ± 0.34	0.222
LDL, mmol/L	2.70 ± 0.86	2.73 ± 0.89	2.65 ± 0.77	0.325
TG, mmol/L	1.96 ± 1.85	1.95 ± 1.75	1.20 ± 2.06	0.784
TC, mmol/L	4.62 ± 1.058	4.65 ± 1.07	4.56 ± 1.02	0.338
BUN/Cr	17.44 ± 6.49	17.63 ± 6.59	17.01 ± 6.25	0.297
UA, μmol/L	327.33 ± 113.71	322.81 ± 107.50	337.88 ± 126.74	0.182

*Data are shown as mean (SD) for continuous variables and as percentages for categorical variables*.

*AIS, acute ischemic stroke; IVT, intravenous thrombolysis; ICH, intracranial hemorrhage; NHISS, National Institute Health of Stroke Scale; SBP, systolic blood pressure; DBP, diastolic blood pressure; AF, atrial fibrillation; APTT, activated partial thromboplastin time; INR, international normalized ratio; PLT, blood platelet; ALB, albumin; WBC, white blood cell; NLR, neutrophil-to-lymphocyte ratio; HDL, High-density lipoprotein; LDL, low-density lipoprotein; TG, triglyceride; TC, total cholesterol; BUN/Cr, blood urea nitrogen-to-creatinine ratio; UA, uric acid*.

### Variable Selection

**Figure 2:**
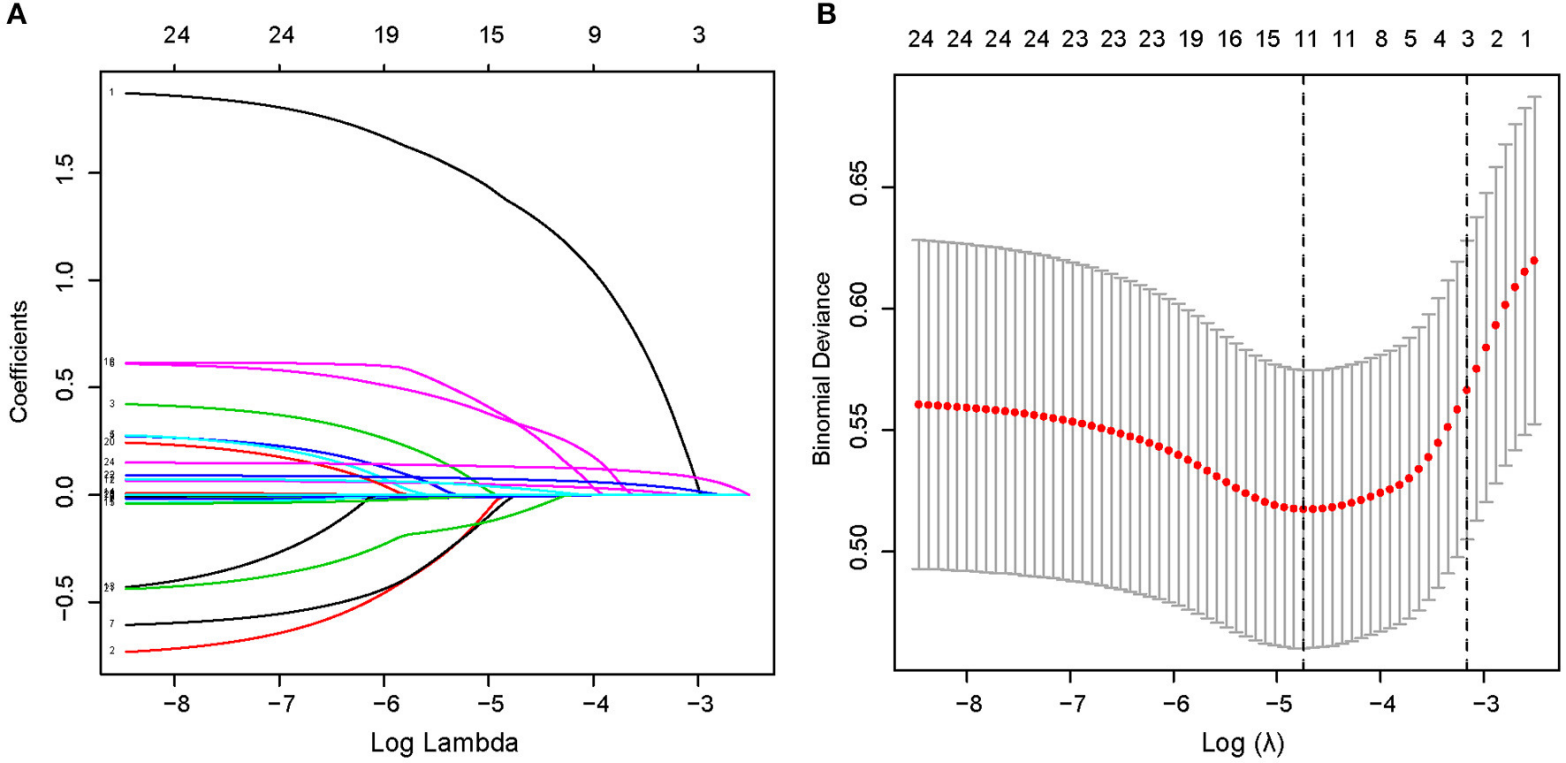
Demographic and clinical feature selection using the LASSO binary logistic regression model in a total of 553 patients. **(A)** The selection of the best parameter (lambda) in the LASSO model uses five-fold cross-validation with the lowest standard. The relationship curve between partial likelihood deviation (binomial deviation) and log(lambda) was plotted. Dotted vertical lines were drawn at the optimal values by using the minimum criteria and the 1 SE of the minimum criteria (the 1—SE criteria). **(B)** LASSO coefficient profiles of the 11 features. A coefficient profile plot was produced against the log(lambda) sequence. A vertical line was drawn at the value selected using five-fold cross-validation, where optimal lambda resulted in five features with non-zero coefficients. LASSO, least absolute shrinkage and selection operator; SE, standard error.

Through searching and analysis of the related literature, 24 potential risk factors among the demographic, clinical, and laboratory indicators of the patients were included in the LASSO regression analysis (Figure 2). The variables with non-zero coefficients in the LASSO regression model were considered to be related to ICH and were selected for further analysis; they included smoking, AF, SBP, DBP, NIHSS, PLT, ALB, HDL, LDL, BUN/Cr, and NLR (Table 2).

**Table 2:** Coefficients and lambda.min value of the LASSO regression.

**Factors**	**Coefficients**	**Lambda.min**
Smoking	1.348	0.009
AF	0.334	
SBP	−0.004	
DBP	−0.008	
NIHSS	0.454	
PLT	−0.001	
ALB	0.031	
HDL	0.336	
LDL	−0.088	
BUN/Cr	0.072	
NLR	0.131	

*AF, atrial fibrillation; SBP, systolic blood pressure; DBP, diastolic blood pressure; NHISS, National Institute Health of Stroke Scale; PLT, blood platelet; ALB, albumin; HDL, High-density lipoprotein; LDL, low-density lipoprotein; BUN/Cr, blood urea nitrogen-to-creatinine ratio; NLR, neutrophil-to-lymphocyte ratio*.

### Univariable and Multivariable Analyses

A univariable analysis of the training set revealed that smoking, AF, NIHSS, BUN/Cr, and NLR were related to ICH. These factors were therefore used in multivariable logistic regression analysis for screening independent clinical predictors of ICH. Multivariable logistic regression analysis demonstrated that four variables (smoking, NIHSS, BUN/Cr, and NLR) are independently associated with ICH (*p* < 0.05), as shown in Table 3. The results indicated that these four variables were independent clinical predictors of ICH in patients with AIS after IVT.

**Table 3:** Univariable and multivariable analyses of intracranial hemorrhage in AIS patients with Intravenous thrombolysis in the training set.

**Variables**	**Univariable analysis**			**Multivariable analysis**		
	**OR**	**95% CI**	***P*-value**	**OR**	**95% CI**	***P*-value**
Smoking	5.874	2.712–12.704	<0.001	9.891	3.875-26.771	<0.001
AF	3.522	1.383–8.255	0.005	2.205	0.718-6.212	0.146
SBP	0.987	0.971–1.004	0.138			
DBP	0.982	0.954–1.012	0.234			
NIHSS	1.089	1.040–1.139	<0.001	1.082	1.022–1.148	0.007
PLT	0.995	0.987–1.001	0.137			
ALB	1.060	0.965–1.168	0.233			
HDL	2.304	0.859–5.916	0.088			
LDL	0.638	0.388–1.009	0.067			
BUN/Cr	1.116	1.065–1.172	<0.001	1.110	1.051–1.176	<0.001
NLR	1.214	1.117–1.330	<0.001	1.151	1.047–1.268	0.003

*CI, confidence interval; OR, odds ratio; AF, atrial fibrillation; SBP, systolic blood pressure; DBP, diastolic blood pressure; NHISS, National Institute Health of Stroke Scale; PLT, blood platelet; ALB, albumin; HDL, High-density lipoprotein; LDL, low-density lipoprotein; BUN/Cr, blood urea nitrogen-to-creatinine ratio; NLR, neutrophil-to-lymphocyte ratio*.

### Predictive Model Development

**Figure 3:**
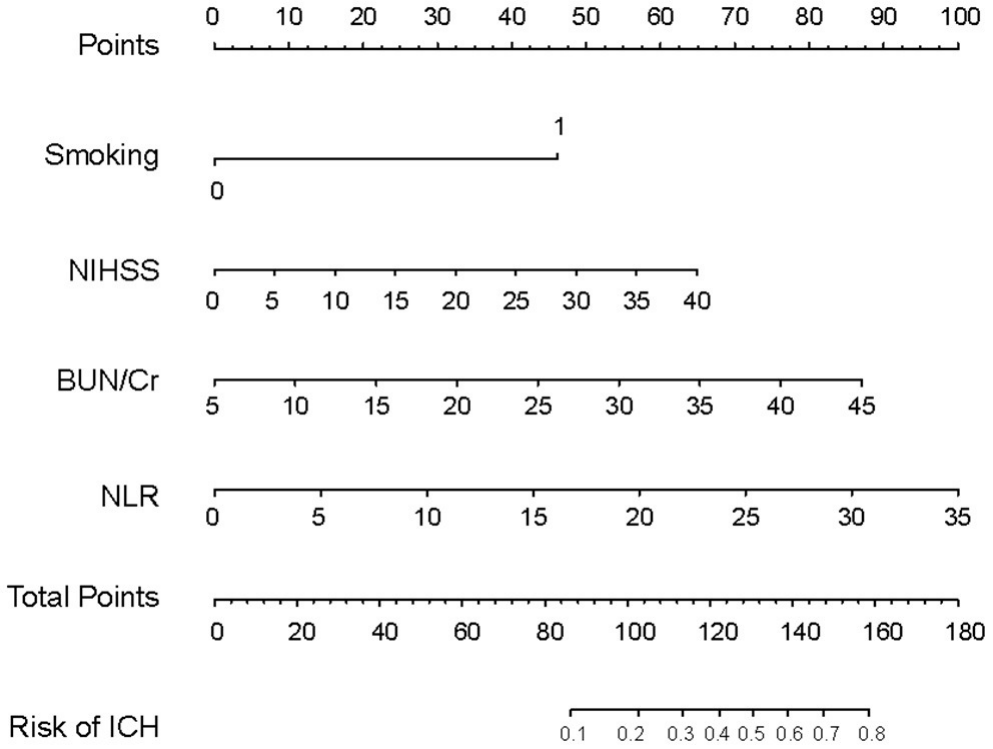
Nomogram model for predicting individual risk of intracranial hemorrhage in AIS patients with intravenous thrombolysis. For all patients, adding up the points identified on the points scale for all four indicators. Then, the sum is located on the “Total Points” axis. Finally, the risk of ICH according to the nomogram is the probability of “ICH” corresponding to “Total Points”. NHISS, National Institute Health of Stroke Scale; BUN/Cr, blood urea nitrogen-to-creatinine ratio; NLR, neutrophil-to-lymphocyte ratio.

The four variables selected by logistic regression analysis (smoking, NIHSS, BUN/Cr, and NLR) were used to build a nomogram for predicting the risk of ICH in AIS patients with IVT (Figure 3). The ratios of the calculated beta were used to evaluate the proportional prognostic effects of these variables. The projections from total points on the scales below indicated the estimated probability of ICH.

### Nomogram Validation

**Figure 4:**
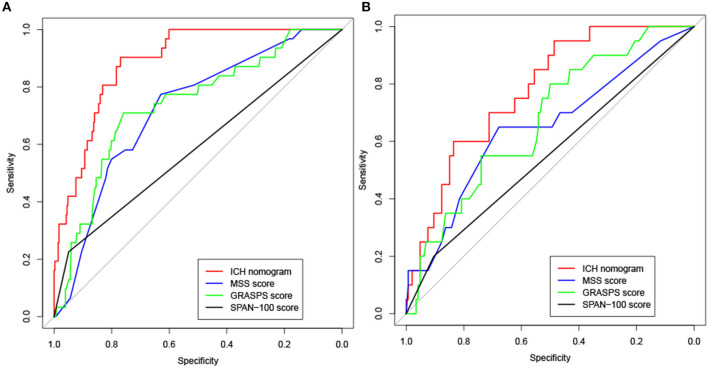
Receiver operating characteristic (ROC) curve analysis for the ICH nomogram, MSS scores, GRASPS scores, and SPAN-100 scores in the training set **(A)** and testing set **(B)**. MSS (Multicenter Stroke Survey); GRASPS (Glucose, Race, Age, Sex, Systolic blood Pressure, and Severity of stroke); SPAN-100 (stroke prognostication using age and NIH Stroke Scale−100 positive index).

To assess the performance of the nomogram in predicting ICH, the likelihood that each patient would experience ICH was also predicted based on his or her MSS scores, GRASPS scores, and SPAN-100 scores. We compared the discrimination of our nomogram with those obtained using MSS scores, GRASPS scores, and SPAN-100 scores using AUC-ROC. As shown in Figure 4 and Table 4, the value of AUC-ROC obtained using our nomogram (0.887; 95% CI: 0.842–0.933) is greater than that obtained using the MSS scores (0.723; 95% CI: 0.637–0.808), the GRASPS scores (0.738; 95% CI: 0.646–0.831), and the SPAN-100 scores (0.588; 95% CI: 0.512–0.663) in the training set (*p* < 0.05). Similar to the results obtained with the training set, the value of AUC-ROC in our model (0.776; 95% CI: 0.681–0.872) was superior to the values obtained using the MSS scores (0.647; 95% CI: 0.508–0.785), the GRASPS scores (0.671; 95% CI: 0.552–0.791), and the SPAN-100 scores (0.552; 95% CI: 0.459–0.645) in the testing set (*p* < 0.05).

**Table 4:** The comparison of AUC-ROC of the ICH nomogram, MSS scores, and GRASPS scores for predicting the risk of ICH in the training set and testing set.

	**The training set**			**The testing set**		
	**AUC-ROC**	**95%CI**	*P-*value^*^	**AUC-ROC**	**95%CI**	*P-*value^*^
ICH nomogram	0.887	0.842–0.933		0.776	0.681–0.872	
MSS score	0.723	0.637–0.808	<0.01	0.647	0.508–0.785	<0.05
GRASPS score	0.738	0.646–0.831	<0.01	0.671	0.552–0.791	<0.05
SPAN-100 score	0.588	0.512–0.663	<0.01	0.552	0.459–0.645	<0.01

*CI, confidence interval; MSS (Multicenter Stroke Survey); GRASPS (Glucose, Race, Age, Sex, Systolic blood Pressure, and Severity of stroke), SPAN-100 (stroke prognostication using age and NIH Stroke Scale−100 positive index)*.

* *DeLong's test was used to compare the differences of AUC-ROC between ICH nomogram and MSS score, GRASPS score and SPAN-100 score. The p < 0.05 was considered statistically significant*.

**Figure 5:**
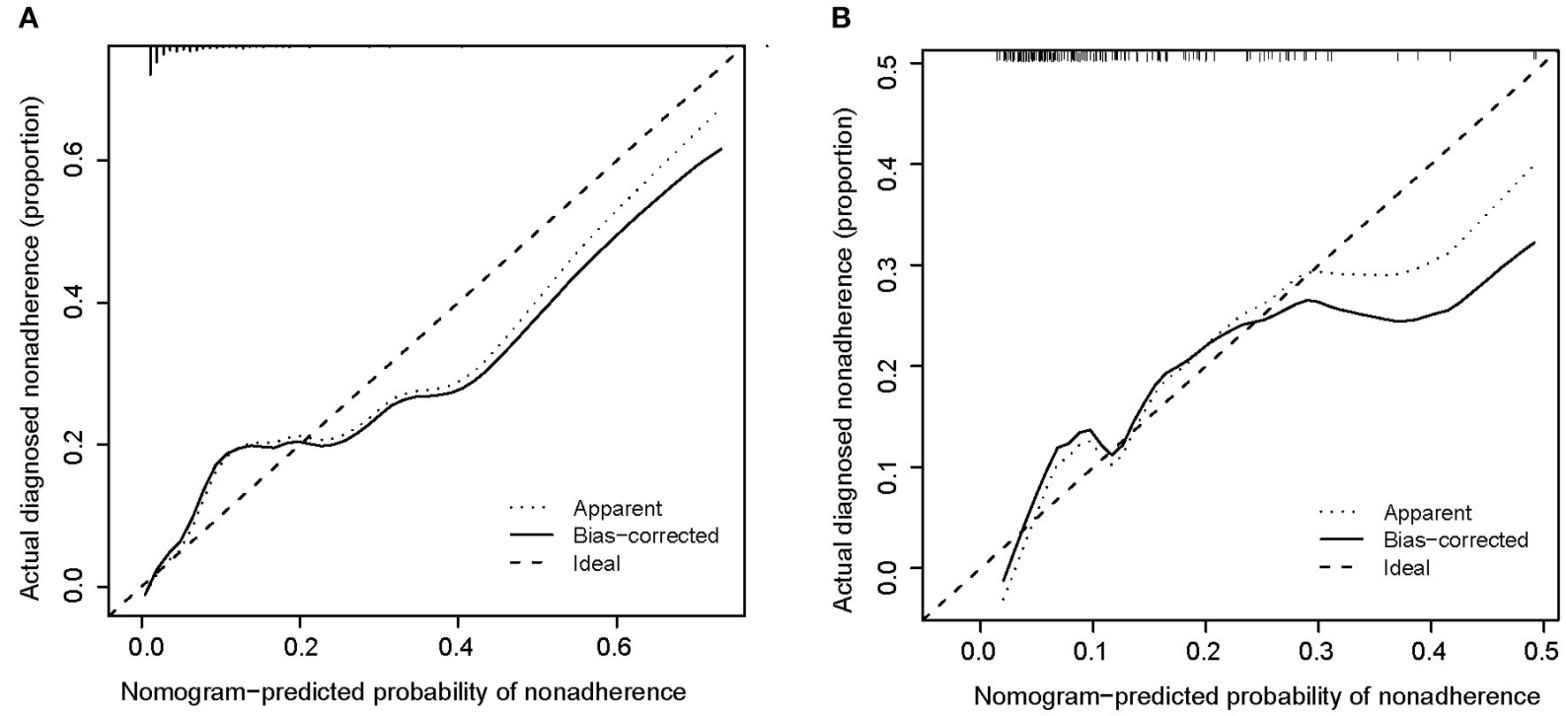
Calibration curve of the nomogram for the training set **(A)** and the testing set **(B)**. Training set: *B* = 1,000 repetitions, boot, mean absolute error = 0.025, *n* = 387; testing set: *B* = 1,000 repetitions, boot, mean absolute error = 0.026, *n* = 166.

**Figure 6:**
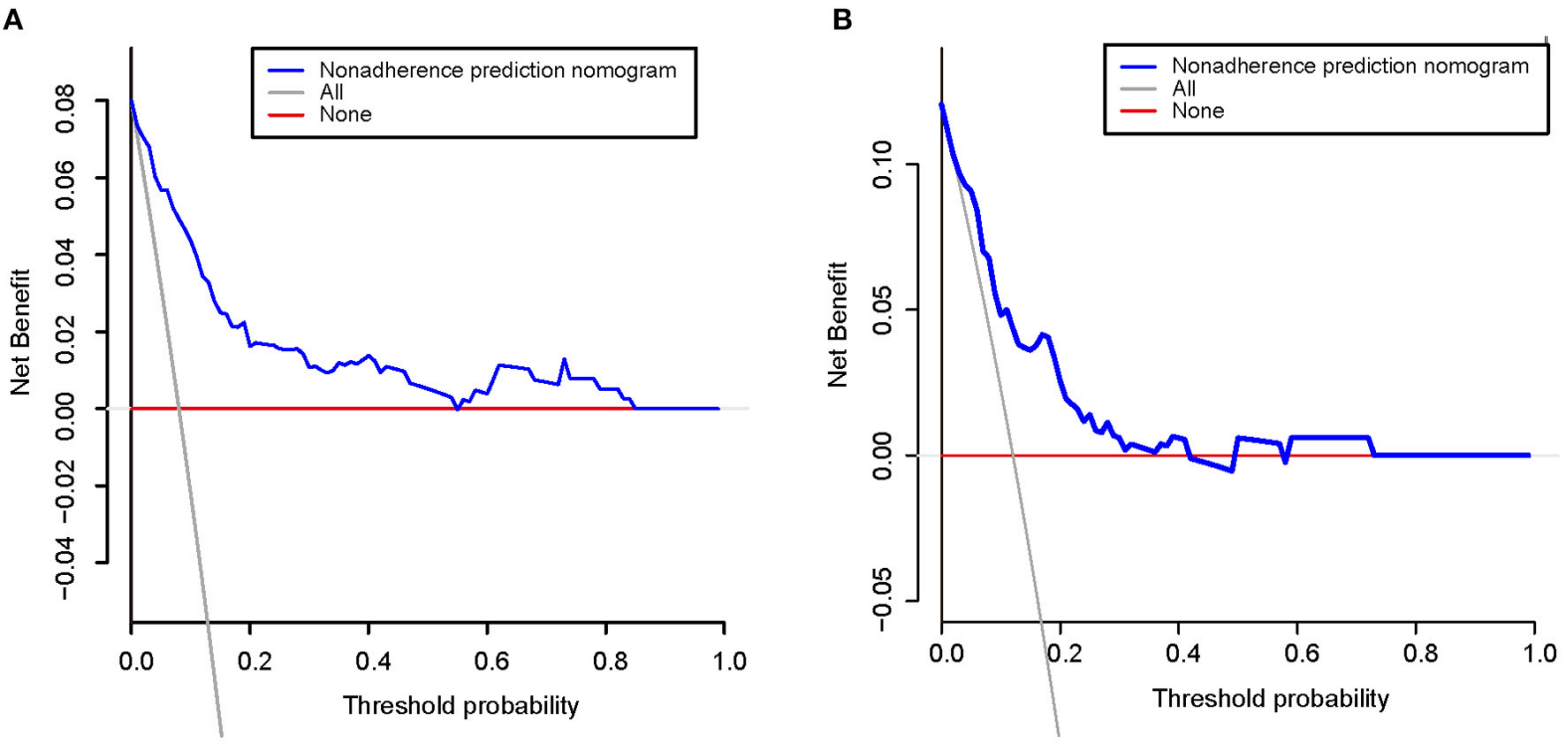
Decision curve analysis for the training set **(A)** and the testing set **(B)**. A horizontal line indicates that all samples are negative and not treated, with a net benefit of zero. An oblique line indicates that all samples are positive. The net benefit is a backslash with a negative slope.

Figures 5A,B shows calibration plots for the training and testing sets. The calibration plot for the training set showed excellent concordance between the predicted probability of ICH and the actual observations; the mean absolute error was 0.024. The calibration plot for the testing set also showed optimal agreement; there, the mean absolute error was 0.036. As shown in Figure 6, DCA suggests that the threshold probabilities ranged from 2.5 to 57.8% in the training set and from 5.4 to 38.2% in the testing set, indicating that the nomogram is clinically useful.

## Discussion

We developed a new nomogram based upon smoking, NIHSS scores, BUN/Cr, and NLR for use in predicting the risk of ICH after IVT therapy. The developed nomogram demonstrated good discrimination and calibration in the training and testing sets. Furthermore, the AUC-ROC curve obtained using our nomogram showed better performance than the curves obtained using the MSS scores, the GRASPS scores, and the SPAN-100 score in both the training and testing sets. In addition, the DCA results suggested that the developed nomogram has a marked net benefit for predicting the risk of ICH.

Six scoring systems for predicting the risk of ICH in patients with AIS after IVT have been proposed. However, most of these scoring systems for individualized prediction of ICH are limited. The blood sugar, early infarct signs, hyperdense cerebral artery sign, age, NIH Stroke Scale (SEDAN) scores and the hemorrhage after thrombolysis (HAT) scores rely on CT images to detect early ischemic changes and hyperdense cerebral artery signs (6, 7). However, those scoring systems have some disadvantages. On the one hand, performing imaging tests is time-consuming, and this could delay the diagnosis and treatment of AIS with a high risk of ICH. On the other hand, hospitals in poor areas or poor communities lack imaging equipment, so these scores may not be suitable for use in patients in these situations. The Safe Implementation of Treatment in Stroke-Symptomatic IntraCerebral Hemorrhage (SITS-SICH) risk score requires data on the patient's history of antiplatelet therapy (8). Because we did not collect data on antiplatelet therapy in the present study, it is difficult to compare the performance of our nomogram with that of the SITS-SICH score. To assess the performance of the nomogram model compared to that of other scoring systems, the AUC-ROC curve was used to compare the prediction accuracy of our model with those of the MSS scores, the GRASPS scores, and the SPAN-100 scores (9, 10, 16). The AUC-ROC value of our predictive nomogram was superior to that of the MSS scores, the GRASPS scores, and the SPAN-100 scores in the training set and the testing set (*p* < 0.05). The better performance of our nomogram may be explained as follows. First, the three scoring systems with which it was compared converted continuous variables to dichotomization/categorization variables; this conversion is statistically inefficient and may decrease the accuracy of prediction (20). Second, the other scoring systems do not include inflammation-related indicators, such as neutrophils, lymphocytes, and NLR, despite the fact that several research studies have shown that the inflammatory response plays a key role in patients with AIS after IVT (21, 22). In addition, many studies have shown that renal dysfunction is related to the outcome of patients with AIS treated with IVT, but the other scoring systems do not take renal function into consideration (23–25).

In addition to the three scoring systems discussed above, three studies designed nomograms to predict the risk of ICH in AIS patients treated with rt-PA. STARTING-SICH (systolic blood pressure, age, onset-to-treatment time for thrombolysis, NIHSS score, glucose, aspirin alone, aspirin plus clopidogrel, anticoagulant with INR ≤1.7, current infarction sign, hyperdense artery sign) nomogram was constructed to predict ICH in stroke patients after IVT treatment in Italy (14). However, this nomogram has not been externally validated for Asian patients, for whom the risk of ICH is higher than for Caucasians. In addition, some of the risk factors included in the STARTING-SICH nomogram are not easily acquired on admission. The other two nomograms were developed in Asian populations (12, 17). Those nomograms take into account only demographic and clinical factors, such as hypertension, blood glucose, PLT, and NIHSS scores, and ignore some of the information that can be acquired from laboratory examination and may reflect risk factors for predicting ICH. In addition, in our study, the AUC-ROC value obtained using the nomogram constructed by Zhou (12) was 0.663 in the training set and 0.662 in the testing set. When the AUC-ROC is <0.700, the discriminative power of the test is not statistically significant (26–28). The other nomogram comprised three variables: AF, NIHSS scores, and glucose level (17). However, the number of cases in which this nomogram was evaluated was relatively small (*n* = 345). In addition, the percentage of ICH based on this nomogram was 14.5%, significantly higher than the percentage of ICH in Asian patients (29, 30). These factors reduce the credibility of this research.

In this study, smoking, NIHSS scores, BUN/Cr, and NLR were shown to be independent predictors of ICH in patients with AIS after rt-PA administration. Consistent with previous studies, smoking and NIHSS were common risk factors for predicting ICH. A previous study reported that age ≥68 years, smoking, AF, SP ≥149 mmHg 2 h after rt-PA administration, and NIHSS scores ≥17 before thrombolysis were associated with the risk of ICH after IVT (31). In Wang's study, smoking, prolongation of APTT, low fibrinogen levels, and low platelet counts were related to the risk of ICH (32). Another study also showed that age ≥70 years, cardioembolism, NIHSS ≥20, and serum glucose ≥9.0 mmol/L on admission were independent risk factors for predicting ICH in patients with AIS after rt-PA administration (33). However, few studies have focused on the BUN/Cr ratio or NLR for predicting the probability of ICH in patients with AIS after IVT. In fact, both BUN/Cr and NLR could affect the outcome of AIS. At present, BUN/Cr ≥15 is considered a biomarker of dehydration, especially in patients with normal kidney function (34). Lin's study demonstrated that BUN/Cr may be a novel predictor of early clinical deterioration in patients with AIS (35). Several studies suggested that dehydration status (BUN/Cr ratio ≥ 15) was a predictor of unfavorable long-term poor prognosis in patients with AIS treated with rt-PA (36, 37). A study showed that an increased BUN/Cr ratio indicated that patients suffered from acute kidney injury and acute heart failure (38, 39). At the same time, previous studies have demonstrated that impaired kidney function increases the risk of bleeding and hemorrhagic microangiopathy (40). Therefore, increased BUN/Cr might explain why the risk of ICH increases in AIS patients with IVT.

The NLR is another new risk factor we found that can contribute to the risk of ICH. Neuroinflammation is related to the entire pathological process of stroke. The NLR is a readily accessible and reproduces new inflammatory biomarker (41). It was reported that a high NLR (≥4.255) on admission increases the risk of ICH in patients with AIS after IVT (42). A recent meta-analysis also indicated that the NLR at admission is an independent risk factor for predicting hemorrhagic transformation after IVT (43). The underlying mechanism through which the NLR increases the risk of ICH in patients with AIS who receive IVT has not been elucidated. One reasonable explanation may be that the NLR affects the outcome because it is associated with the inflammatory destruction of neutrophils and a reduced protective effect of lymphocytes (44). Some studies have reported that peripheral neutrophils and T cells stimulated by tissue-type plasminogen activator (tPA) transmigrate to the brain vasculature, where they disrupt the blood-brain barrier (BBB) and accelerate ICH (45, 46).

Generally, the nomogram developed in this study, which includes four parameters, is convenient and efficient for use in the management of patients with AIS. First, the predictors used in our predictive nomogram are easily available at almost all medical centers, even those in poor areas. In addition, the nomogram is particularly suitable for use by non-neurologists because CT imaging is excluded. Furthermore, the discrimination and calibration performance of the nomogram was good; therefore, it could be a reliable tool for predicting the risk of ICH in patients with AIS after rt-PA treatment.

Our study has some limitations. First, our data came from a single-center retrospective analysis, and this might have limited the statistical power of the results. Second, our model has not been validated in external cohorts. Prospective, multicenter studies will be required in the future to assess the applicability of our nomogram. Third, data on onset-to-treatment time for thrombolysis or administration of oral antiplatelet drugs or anticoagulants, all of which may be risk factors for ICH, were not available in our study.

## Conclusion

In conclusion, the new nomogram that includes smoking, NIHSS, BUN/Cr, and NLR may predict the risk of ICH after IVT in AIS patients in the Asian population.

## Data Availability Statement

The raw data supporting the conclusions of this article will be made available by the authors, without undue reservation.

## Ethics Statement

The studies involving human participants were reviewed and approved by the Ethics Committee of the Central Hospital of Shaoyang. Written informed consent for participation was not required for this study in accordance with the national legislation and the institutional requirements.

## Author Contributions

A-DX, DL, Z-AW, and X-XH contributed to the study conception and design. Material preparation, data collection were performed by DD, Z-GY, S-YL, J-KZ, Y-FLi, Y-FLiu, Y-SW, T-YZ, and X-LS. Analysis and interpretation of the data were done by Z-AW. The first draft of the manuscript was written by Z-AW, X-XH, A-DX, and DL. All authors read and approved the final manuscript.

## Funding

This work was supported by grants from the National Natural Science Foundation of China (81671167, 81971121, 81801150, and 82171316), the Science and Technology Planning Project of Guangdong Province, China (2017A020215049 and 2019A050513005), Natural Science Foundation of Guangdong Province (2018A0303130182 and 2020A1515010279), Postdoctoral Research Foundation of China (2018M643370 and 2021M701419), Guangzhou Science and Technology Planning Project (201508020004), Technology and People's Livelihood Major Project of Guangzhou (2014Y2-00505), and the Fundamental Research Funds for the Central Universities (21621102).

## Conflict of Interest

The authors declare that the research was conducted in the absence of any commercial or financial relationships that could be construed as a potential conflict of interest.

## Publisher's Note

All claims expressed in this article are solely those of the authors and do not necessarily represent those of their affiliated organizations, or those of the publisher, the editors and the reviewers. Any product that may be evaluated in this article, or claim that may be made by its manufacturer, is not guaranteed or endorsed by the publisher.

## Abbreviations

AF: atrial fibrillation
AIS: acute ischemic stroke
ALB: albumin
APTT: activated partial thromboplastin time
BUN/Cr: blood urea nitrogen-to-creatinine ratio
DBP: diastolic blood pressure
HDL: High-density lipoprotein
ICH: Intracranial hemorrhage
INR: International normalized ratio
IVT: intravenous thrombolysis
LASSO: The least absolute shrinkage and selection operator
LDL: low-density lipoprotein
NHISS: National Institute Health of Stroke Scale
NLR: neutrophil-to-lymphocyte ratio
PLT: blood platelet
SBP: systolic blood pressure
TC: total cholesterol
TG: triglyceride
UA: uric acid
WBC: white blood cell.
